# Sleep deficiency promotes Alzheimer's disease development and progression

**DOI:** 10.3389/fneur.2022.1053942

**Published:** 2022-12-14

**Authors:** Ya-Nan Lv, Yu Cui, Bo Zhang, Shu-Ming Huang

**Affiliations:** ^1^Department of Neuroscience, Institute of Chinese Medicine, Heilongjiang University of Chinese Medicine, Harbin, China; ^2^Department of Veterinary Medicine, School of Animal Science and Technology, Hainan University, Haikou, China

**Keywords:** sleep deficiency, Alzheimer's disease, β-amyloid protein, tau protein, oxidative stress, inflammatory response, glucocorticoid, BDNF

## Abstract

Sleep disorders are a common health problem in modern society. Long-term sleep deficiency increases the risk for Alzheimer's disease. However, the exact mechanisms by which sleep deficiency affects Alzheimer's disease remain unclear. Therefore, we reviewed the relevant studies and investigated the role of sleep deprivation in Alzheimer's disease pathogenesis. Sleep deficiency was found to be associated with oxidative stress, β-amyloid protein deposition, tau hyperphosphorylation, and neuroinflammation, which are known to increase the risk for Alzheimer's disease. In addition, insufficient sleep also increases glucocorticoid levels, decreases brain-derived neurotrophic factor levels, and reduces the number of synapses in the central nervous system. These factors also promote Alzheimer's disease development and progression. The present study showed that a growing body of evidence supports an association between sleep disturbances and Alzheimer's disease. It discusses the role of sleep insufficiency in Alzheimer's disease pathogenesis, which may provide a theoretical basis for effective treatment and prevention strategies.

## 1. Introduction

Sleep deficiency occurs when the body does not get the required amount of sleep, i.e., insufficient sleep time or poor sleep quality. The earliest research on sleep deficiency can be traced to more than 100 years ago (1). Approximately 38.2% of the general Chinese population has insomnia symptoms (2). Humans store energy during sleep, reverse damages caused while awake, and process information to facilitate learning and memory (3). Sleep deficiency can cause various adverse effects, including reduced reaction time, reduced vigilance, increased perceptual and cognitive distortion, emotional changes, and even neurodegenerative diseases, including Alzheimer's disease (AD) and Parkinson (4). Sleep deficiency is a common early symptom of neurodegenerative diseases. Long-term sleep deficiency affects emotions, learning, and memory (5, 6).

AD is an age-related neurodegenerative disease of the central nervous system characterized by progressive cognitive and memory impairment and the loss of general intelligence, including memory, judgment, and abstract thinking (7). The most characteristic pathological changes associated with AD are senile plaques (SPs) formed by the deposition of β-amyloid protein (Aβ), neurofibrillary tangles (NFTs) formed by intracellular aggregation of abnormally phosphorylated tau proteins, loss of neuronal synapses, and reduced numbers of neurons (8).

Recent studies have extensively investigated the effect of sleep deficiency on AD (9). Clinical and animal experiments suggest that chronic sleep insufficiency may increase AD incidence and accelerate its pathogenesis (10). However, the pathophysiological mechanisms by which long-term sleep deficiency promotes AD progression remain unclear. In this study, we reviewed the literature and analyzed relevant studies to determine the mechanisms for AD induced by long-term sleep deficiency.

## 2. Mechanism of AD caused by sleep deficiency

Studies have shown that long-term insomnia increases the risk for neurodegenerative diseases, including AD (11). Epidemiological investigations have revealed that about 44% of AD patients had sleep disorders and circadian rhythm disorders (12, 13). With increasing age, the circadian rhythm and sleep-wake regulation system of the elderly gradually degenerate, while the regulation function is weakened, which significantly increases the risk of AD (14). A meta-analysis of 27 observational studies showed that the risk for AD increased by 3.78 times with insomnia and that effective insomnia interventions could delay AD progression in about 15% of the patients (15). In addition, studies have shown that non-rapid eye movement (NREM) sleep slow wave activity decreases with increase of Aβ deposition and tau accumulation (16). Roh et al. reported that a normal sleep-wake cycle and diurnal fluctuation of interstitial fluid (ISF) Aβ are present in the brain of APPswe/PS1δE9 mice before Aβ plaque formation. Following plaque formation, the sleep-wake cycle markedly deteriorated and the diurnal fluctuation of ISF Aβ dissipated (17). Therefore, investigating sleep deficiencies could be significant for AD prevention and treatment.

### 2.1. Sleep deficiency promotes Aβ deposition

Preclinical studies have demonstrated that neurons release Aβ in an activity-dependent manner under physiological conditions, and that brain Aβ levels show diurnal fluctuations; secretion increases when awake and decreases during sleep (18). Compared to high-quality rest, decreased, low-quality or slow-wave sleep increases cortical neuronal activity and Aβ release (19). With continuous Aβ plaque formation in sleep-regulation centers, sleep cycle-related variations in extracellular Aβ levels disappear. This creates a positive feedback loop; insufficient sleep leads to Aβ deposition and Aβ plaques further affect sleep (20). Kang et al. found that ISF Aβ levels were correlated with wakefulness using *in vivo* microdialysis, and they demonstrated that Aβ levels in the brain increased and plaque deposition potentially increased in both mouse and human sleep disorders (21). Ooms et al. demonstrated that Aβ1-42 levels in the cerebrospinal fluid increased significantly in healthy males during sleep deprivation, and this change was reversed during good sleep at night, suggesting that short-term sleep deprivation increases Aβ levels (22). Studies in transgenic mice have shown that locus coeruleus degeneration and impaired cortical norepinephrine neuron function could increase the inflammatory response, which was related to increased Aβ and memory deficits (23). Mammalian brain control sleep and wakefulness through complex interactions between subcortical neuromodulatory neurons in the brain stem, midbrain, hypothalamus, and basal forebrain, thalamus, and cortex drive behavioral, physiological, and electrocortical sleep/wake states. Locus coeruleus is also a major brain region among the wake-promoting monoaminergic and cholinergic populations (24). It has also been demonstrated that chronic sleep deficiency increases extracellular Aβ concentration in the brains of model animals, while prolonged sleep reduces Aβ plaque formation (25). Hence, sleep contributes to Aβ clearance, while sleep deprivation promotes Aβ deposition, thus forming the characteristic pathological changes of AD.

### 2.2. Sleep deficiency induces abnormal tau protein phosphorylation

Tau protein is a protein that regulates and maintains microtubule stability. Under normal conditions, the phosphorylation/dephosphorylation level of tau protein is balanced, which promotes microtubule aggregation and maintains its stability (26). Tau hyperphosphorylation leads to its accumulation and formation of pairs of double helix structures (27). In the brains of AD patients, excessive tau phosphorylation and aggregated NFT deposition results in neuronal degeneration and apoptosis (28). NFTs are the primary brain microstructural features of AD. It has been demonstrated that adults with extreme sleep deficiency have increased tau protein levels in the brain and cerebrospinal fluid (29). Evidence from animal models indicates that changes in sleep-wake cycles increase hyperphosphorylated tau protein levels in the brain (30). Holth et al. showed that mouse ISF tau increased ~90% during normal wakefulness vs. sleep and ~100% during sleep deprivation. In humans, tau levels in the cerebrospinal fluid also increased by more than 50% during sleep deprivation. Thus, the sleep-wake cycle regulates tau level in the brain, and sleep deprivation increases cerebral tau and its pathological diffusion (31). It has been reported that sleep deficiency for two consecutive months can lead to >50% increase in insoluble Tau in the brains of AD patients (32). It can be seen that insufficient sleep can lead to increased tau protein levels, thereby increasing the risk of AD. Thus, optimization of sleep-wake cycle is important for the prevention and treatment of AD.

### 2.3. Sleep deficiency increases oxidative stress in the brain

Oxidative stress refers to an imbalance between oxidation and anti-oxidation *in vivo*. Oxidative reactions provide an advantage by producing large numbers of oxidation intermediates (33). Studies have shown that sleep deprivation is linked to free radicals production, which induces oxidative stress. Sleep protects the brain by reducing free radical production (34). The oxidative stress response is influenced by sleep deprivation through three mechanisms. First, sleep deprivation causes abnormal energy metabolism and increases the production of reactive oxygen species and other free radicals. Second, it suppresses the antioxidant defense system. Third, sleep deprivation causes endoplasmic reticulum stress, which indirectly causes oxidative stress (35). In a study, the concentration of glutathione was significantly reduced in rat brains after 96 h of rapid eye movement sleep deprivation compared to controls (36). Ramanathan et al. showed that long-term sleep deprivation significantly decreased the antioxidant activity of superoxide dismutase in rat hippocampi and brainstems (37). Studies have also shown that reduced efficiency of the antioxidant system and excessive production of free radicals, including superoxide anion, hydrogen peroxide, and nitric oxide, are involved in AD pathogenesis (38). The positive correlation between amyloid plaque and lipid peroxidation markers, 4-hydroxynonaldehyde and malondialdehyde (MDA), supports this hypothesis (39). Therefore, insufficient sleep may promote AD by increasing oxidative stress in the brain.

### 2.4. Sleep deficiency induces neuroinflammation

Neuroinflammation occurs in all neurodegenerative diseases and may be involved in their pathogenesis (40). Microglial cells are involved in immune functions and internal environment homeostasis in the brain. Excessive microglial activation releases inflammatory factors and promotes neuroinflammation (41). Long-term sleep deficiency can lead to chronic systemic low-grade inflammation and is associated with various inflammatory diseases (42). In sleep-related studies, limiting the sleep time for healthy participants to 4 h/day for five consecutive days increased plasma interleukin-6 and C-reactive protein levels in most participants (43). This indicates that a non-specific inflammatory response occurs with prolonged sleep deficiency. It was also reported that serum tumor necrosis factor levels decreased during sleep but increased after 2 days of normal sleep, which indicates the regulation inflammatory cytokines by sleep (44). Spangenberg et al. suggested that extracellular Aβ accumulation may cause chronic neuroinflammation in AD and proposed a microglia-mediated chronic neuroinflammation model, which showed that Aβ binds to microglial toll-like receptors during AD development (45). Initial microglial activation develops into chronic inflammation due to continued stimulation, leading to reduced synaptic remodeling and neuronal death (46). Therefore, inflammation is hypothesized to be a biologically plausible pathway linking sleep disturbance and the risk of AD.

### 2.5. Sleep deficiency increases glucocorticoid levels

Physiological glucocorticoids regulate growth, immunity, and metabolism (47). When the body encounters injury or stress, excessive glucocorticoids can exert negative effects (48). Long-term stress leads to the dysfunction of hypothalamic-pituitary-adrenal (HPA) axis, resulting in a sustained increase in blood glucocorticoid levels (49).

Clinical studies have shown that cortisol levels increase in the early stages of AD (50). Excessive cortisol secretion may promote neuronal loss and accelerate cognitive decline and disease progression. A longitudinal study from Baltimore suggests that elevated cortisol levels may increase the risk for AD in the elderly (51). Animal experiments have shown that long-term sleep deprivation reduces cell proliferation and adult neurogenesis in rat dentate gyri by increasing glucocorticoids (52). Therefore, increased glucocorticoid levels caused by insufficient sleep lead to decreased cell proliferation. It has been reported that HPA axis hyperactivity may be related to chronic insomnia (53) and that sleep interruptions may be caused by increased corticotropin-releasing hormone (54). Studies have also shown that glucocorticoid receptor antagonists improve sleep quality and may be used to treat chronic insomnia by regulating HPA axis activity (55). Glucocorticoid upregulation is also a typical pathological feature of these two conditions.

### 2.6. Sleep deficiency reduces synaptic plasticity

Synapses form connections between neurons and are essential for information transmission (56). Synaptic plasticity, including structural and functional plasticity, is a primary manifestation of neural plasticity, which reflects the variability in synaptic morphology, function, and number (57). Sleep deficiency reduces synaptic plasticity, which impairs learning and memory and increases the risk of cognitive impairment in insomniac individuals (58). Studies have shown that synaptic astrocytes promote the development and maturation of dendritic spines and regulate synaptic plasticity. Sleep deficiency reduces dendritic spine density by inhibiting hippocampal astrocytes regulation, thereby affecting the normal hippocampal function (59). A recent positron emission tomography imaging study of AD patients found that sleep deprivation significantly reduced hippocampal synaptic density (60). Wang et al. demonstrated that chronic sleep deprivation aggravated hippocampal synaptic plasticity damage in APP/PS1 double transgenic AD model mice (61). Thus, insufficient sleep decreases the number of synapses in AD patients and impairs signal transduction between neurons.

### 2.7. Sleep deficiency affects brain-derived neurotrophic factor levels

Brain-derived neurotrophic factor (BDNF), a neurotrophic protein synthesized in the brain, promotes neuronal growth, development, survival, and differentiation (62). It is an important regulator of learning and memory (63). BDNF is down-regulated in both AD and sleep deficiency and is, therefore, a common pathological feature.

Synaptic plasticity in p75 neurotrophin receptor gene knock-out mice after sleep deprivation was found to depend on the enhancement of BDNF pathway conduction (64). Increased expression of BDNF, postsynaptic density protein 95, and other synaptic plasticity-related proteins significantly alleviates hippocampal memory and learning disorders caused by sleep deficiency (65). Animal experiments have also shown that the hippocampal BDNF expression increased 24 h after acute sleep deprivation in mice (66). This was consistent with previous studies that reported that short-term sleep deprivation in humans up-regulated BDNF levels (67). In animal experiments, BDNF consumption and loss increased the number and size of cortical amyloid plaques and aggravated the neuropathological changes in AD mice (68). Increased BDNF levels may also reduce abnormal Aβ production (69).

## 3. Summary

**Figure 1:**
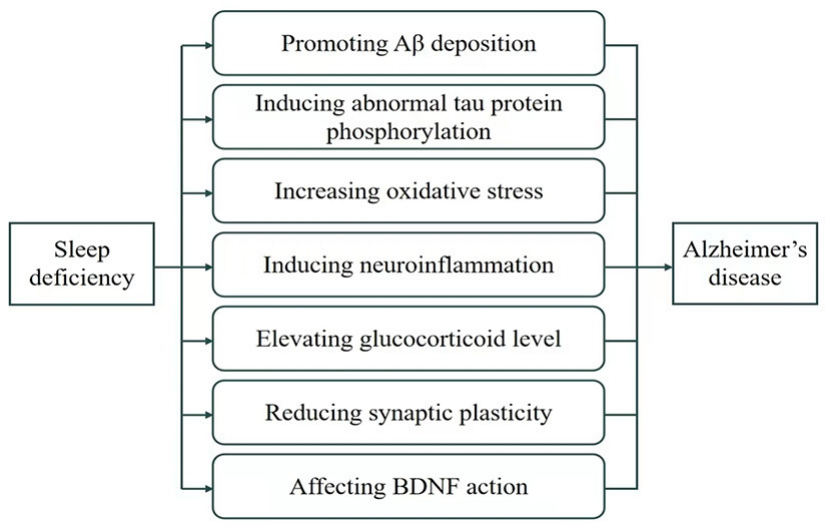
Mechanism summary of sleep deficiency and AD.

A growing body of evidence has demonstrated a close relationship between sleep deficiency and AD. Sleep deficiency induces and aggravates AD development and progression (15). Sleep insufficiency accelerates Aβ generation and deposition, promotes Tau protein hyperphosphorylation, and causes oxidative stress and inflammation in the nervous system, thereby increasing the risk for AD. It also reduces the number and transmission function of synapses, increases glucocorticoid level, and decreases BDNF levels, which further promotes AD (Figure 1). Therefore, improving sleep quality may be effective in preventing AD progression.

Sleep deficiency is increasingly viewed as an early event in the course of AD. Understanding the mechanisms underlying the effect of sleep deficiency on AD has the potential to optimize efforts for the identification of targets for overcoming AD. This review provides a new perspective for future research on AD, it is that, improving sleep may become an effective means to delay or reduce the occurrence of AD. Therefore, it may provide new insights and entry for researchers to prevent and treat AD by improving sleep in basic and clinical research. Given the evidence in the paper that sleep deficiency is associated with several risk factors for AD, further research is needed to explore how to target improvement of sleep as a novel treatment and even a prevention strategy for AD. We believe that further research on the underlying mechanisms for the association of sleep deficiency with AD will bring us new knowledge.

## Author contributions

Y-NL wrote the manuscript. YC wrote and revised the manuscript. S-MH and BZ provided critical comments and revised the manuscript. All the authors approved the final draft and agreed to be accountable for all aspects of the work.
